# 达雷妥尤单抗联合DECP方案治疗髓外复发多发性骨髓瘤一例

**DOI:** 10.3760/cma.j.issn.0253-2727.2021.08.016

**Published:** 2021-08

**Authors:** 中玲 韦, 来全 黄, 家炜 严, 辰 黄, 然 王, 君 黄, 东平 黄

**Affiliations:** 皖南医学院第一附属医院弋矶山医院，芜湖 241001 Department of Hematology, the Affiliated Yijishan Hospital of Wannan Medical College, Wuhu 241001, China

患者，男，56岁，2019年7月外伤后腰背部持续性疼痛伴活动受限，于当地医院行X线检查示T10椎体压缩性骨折，2019年10月以“多发性骨髓瘤”收入我院。查体：体温36.4 °C，贫血貌，浅表淋巴结未触及肿大，左侧胸廓有压痛，腰椎棘突无压痛及叩击痛。血常规：WBC 5.8×10^9^/L，HGB 67 g/L，PLT 41×10^9^/L；生化常规：白蛋白21.3 g/L，球蛋白138.30 g/L，血钙1.97 mmol/L，肌酐70.7 µmol/L；β_2_-微球蛋白1.3 mg/L；血清蛋白电泳：IgG 157 g/L，IgA 0.078 g/L，IgM 0.124 g/L，κ 0.908 g/L，λ 251.0 g/L，M蛋白126.9 g/L。血清免疫固定电泳提示IgG-λ型M蛋白血症。血清游离κ/λ 0.006，尿液游离κ/λ 0.705。骨髓象提示浆细胞分类占50％；免疫分型可见约27％的异常细胞群体，表达CD27、CD38、CD56、cλ。骨髓活检提示异常浆细胞增生。FISH结果示1q21位点信号扩增（>3拷贝），阳性率约为12％；存在t（4;14），阳性率约为13％，IGH拷贝数增加，比例为11％～12％。染色体核型分析未见分裂象。PET-CT示：T10椎体压缩性骨折，T10前方见软组织肿块，直径约2.8 cm×1.4 cm，左前第四肋骨处见软组织肿块。全身骨多发骨质破坏伴FDG代谢增高（伴左侧第4、5肋骨病理性骨折）。患者诊断为活动性/有症状多发性骨髓瘤IgG-λ型伴髓外病变，DS分期ⅢA期；ISS分期Ⅱ期；R-ISS分期Ⅱ期；IMWG高危；mSMART3.0分期显示“双打击”。予RVD方案（硼替佐米+来那度胺+地塞米松）诱导化疗2个疗程。再次复查骨髓象提示浆细胞占4％，流式细胞术浆细胞检测可见约1.11％残留浆细胞；球蛋白56.10 g/L；血清蛋白电泳提示M蛋白22.14 g/L，血清游离κ/λ 0.092；PET/CT提示左侧第四肋骨软组织肿块消失，（T8-L1水平）脊柱旁高代谢肿块，直径约7.3 cm×5.3 cm，FDG代谢增高。在CT引导下行脊柱旁肿块穿刺病理检查，穿刺物病理结果提示：（穿刺组织）浆细胞瘤；免疫组化结果：CD20（−），CD79a（+/−），CD3（−），CD43（−），CD38（+），CD138（+），MUM-1（+），λ（+），κ（−），Ki-67（40％+），疗效评判为疾病进展。2020年1月开始予达雷妥尤单抗（16 mg/kg，第0、7、14、21天）联合DECP方案（顺铂10 mg/m^2^，第1～4天；环磷酰胺400 mg/m^2^，第1～4天；依托泊苷40 mg/m^2^，第1～4天；脂质体阿霉素40 mg，第2天；28 d为一个周期）挽救化疗2个疗程。化疗结束再次复查PET/CT提示（T8-L1水平）脊柱旁高代谢肿块消失，重新评估疗效达到非常好的部分缓解。2020年4月予依托泊苷（1.6 g/m^2^，第1天）联合G-CSF（7.5 µg/kg，第3天开始）方案成功完成了外周血造血干细胞动员和采集。采集到外周血干细胞总有核细胞数为5.584×10^8^/kg，CD34^+^细胞数为2.046×10^6^/kg。2020年5月，患者再次出现骨痛，复查骨髓象提示浆细胞占29％，PET/CT提示全身多发骨质破坏伴部分FDG代谢增高，左侧肋骨部位见软组织影，T8-11椎体边缘软组织影伴FDG代谢增高。FISH检测提示1q21扩增，阳性率约为18％，存在t（4;14），阳性率约为20％；染色体核型结果48, XY, +add（1）（p36.3）, −2, +3, del（7）（q22）, add（11）（p15）, −14, add（16）（p13.3）,+17, psudic（19;1）（q13;p11）, −20, +3-5mar［cp4］/46, XY[6]。后患者转至外院进行CAR-T细胞治疗无效死亡。

